# Validity of Empatica E4 Wristband for Detection of Autonomic Dysfunction Compared to Established Laboratory Testing

**DOI:** 10.3390/diagnostics15202604

**Published:** 2025-10-16

**Authors:** Jenny Stritzelberger, Marie Kirmse, Matthias C. Borutta, Stephanie Gollwitzer, Caroline Reindl, Tamara M. Welte, Hajo M. Hamer, Julia Koehn

**Affiliations:** 1 Epilepsy Center, Department of Neurology, Friedrich-Alexander-University Erlangen-Nürnberg (FAU), 91054 Erlangen, Germanymatthias.borutta@uk-erlangen.de (M.C.B.);; 2 Department of Neurology, ANregiomed, Escherichstraße 1, 91522 Ansbach, Germany

**Keywords:** autonomic dysfunction, epilepsy, wearable devices, Empatica E4 wristband

## Abstract

**Background:** Heart rate variability (HRV) is a well-established marker of autonomic nervous system (ANS) activity. It is also an important tool for investigating cardiovascular and neurological health. Changes in HRV have been associated with epilepsy and sudden unexpected death in epilepsy (SUDEP), conditions in which autonomic dysregulation is believed to play a significant role. HRV is traditionally measured using electrocardiography (ECG) under standardized laboratory conditions. Recently, however, wearable devices such as the Empatica E4 wristband have emerged as promising tools for continuous, noninvasive HRV monitoring in real-life, ambulatory, and clinical settings where laboratory infrastructure may be lacking. **Methods:** We evaluated the validity and clinical utility of the Empatica E4 wristband in two cohorts. In the first cohort of healthy controls (n = 29), we compared HRV measures obtained with the E4 against those obtained with a gold-standard laboratory ECG device under seated rest and metronomic breathing conditions. In persons with epilepsy (PWE, n = 42), we assessed HRV across wake and sleep states, as well as during exposure to sodium channel blockers. This was done to determine whether the device could detect physiologically and clinically meaningful changes in autonomic nervous system (ANS) function. **Results:** In healthy participants, the Empatica E4 provided heart rate (HR), root mean square of successive R-R intervals (RMSSD), and standard deviation of all interbeat intervals (SDNN) values that were strongly correlated with laboratory measurements. Both devices detected the expected increase in RMSSD during metronomic breathing; however, the E4 consistently reported higher absolute values than the ECG. In patients with epilepsy (PWE), the E4 reliably captured parasympathetic activation during sleep and detected a significant reduction in heart rate variability (HRV) in patients taking sodium channel blockers, demonstrating its sensitivity to clinically relevant autonomic changes. **Conclusions:** The Empatica E4 wristband is valid for measuring HRV in research and clinical contexts. It can detect modulations of ANS activity that are physiologically meaningful. While HRV metrics were robust, other signals, such as electrodermal activity and temperature, were less reliable. These results highlight the potential of wearable devices as practical alternatives to laboratory-based autonomic testing, especially in emergency and resource-limited settings, and emphasize their importance in epilepsy care risk assessment.

## 1. Introduction

Epilepsy is one of the most common chronic neurological disorders in Germany. It is associated with significantly increased mortality rates [1], to which sudden unexpected death (SUDEP) contributes significantly in persons with epilepsy (PWE) [2]. Epileptic seizures are often associated with changes in heart rate and autonomic nervous system (ANS) modulation [3]. Beyond ictal changes, the role of interictal dysregulation of the ANS in PWE is under discussion [4], and autonomic dysfunction is thought to play a causal role in SUDEP [5,6].

Heart rate variability (HRV) is a well-established method of quantifying ANS function. It is frequently used in clinical and research settings to evaluate cardiovascular and autonomic regulation [7]. Traditionally, HRV is derived from electrocardiography (ECG) under standardized laboratory conditions. However, the growing availability of wearable devices offers new opportunities for ambulatory, noninvasive, continuous HRV monitoring in real-world environments.

The Empatica E4 wristband is a commercially available wearable device that records photoplethysmography (PPG)-derived interbeat intervals (IBIs), heart rate (HR), temperature, electrodermal activity (EDA), and movement. Several studies have shown that the E4 can provide pulse rate (PR) and pulse rate variability (PRV) metrics that approximate heart rate (HR) and heart rate variability (HRV) obtained from electrocardiography (ECG) under low-activity conditions [8,9,10]. Recent findings further indicate that the E4 can accurately estimate PR even in short recording windows, underscoring its potential feasibility for clinical application [11].

Such wearable devices are of particular interest in epilepsy, where autonomic dysfunction is both a marker of disease burden and a potential mechanism of SUDEP, and where continuous monitoring across different states is of clinical interest. In particular, sleep represents a physiologically dynamic period with distinct autonomic profiles, and several studies have linked impaired nocturnal ANS regulation to SUDEP risk [12,13]. Likewise, antiseizure medications (ASM), especially sodium channel blockers, have been associated with autonomic side effects that may contribute to cardiovascular risk [14]. Many hospitals lack the infrastructure for comprehensive autonomic testing, and emergency settings often require rapid, unobtrusive assessments. In these contexts, the E4 wristband may represent a valuable alternative, offering portable, real-time ANS monitoring.

Demonstrating that the Empatica E4 can detect clinically relevant changes in autonomic activity is a critical step towards proving its usefulness as an accessible and practical tool for autonomic nervous system (ANS) monitoring in people with epilepsy. Wearable devices such as the E4 could bridge the gap between highly standardised, resource-intensive laboratory assessments and the need for continuous, real-world monitoring in clinical and emergency settings. This study seeks to provide evidence for the integration of wearable devices like the E4 into epilepsy care beyond seizure detection and research by confirming that it can capture changes in autonomic function that are physiologically and clinically meaningful.

Specifically, the study has three main objectives: (I) To validate E4-derived heart rate (HR) and heart rate variability (HRV) parameters against the gold standard for laboratory measurements of autonomic modulation during resting baseline conditions and controlled metronomic breathing, which enhances parasympathetic activity selectively; (II) to characterize autonomic function in people with epilepsy (PWE) across different vigilance states, including wakefulness and sleep, where distinct autonomic patterns are expected; and (III) to investigate the impact of sodium-channel-blocking antiseizure medications on autonomic parameters, given their known influence on cardiovascular regulation.

## 2. Materials and Methods

### 2.1. Participants

29 healthy controls (10 women, 19 men; mean age = 35.8 ± 11.5 years) and 42 patients with epilepsy (20 women, 22 men; mean age = 32.3 ± 13.1 years) participated in the study. Since this was an exploratory study, no a priori sample size or power calculation was performed. Recruitment was constrained by clinical availability and feasibility within the monitoring period. Our primary aim was to validate the device and detect clinically meaningful within-subject and between-condition effects, rather than to test a single pre-specified effect size. Patients were recruited upon admission for long-term video EEG monitoring at the Epilepsy Center, screened for eligibility, and enrolled after providing written informed consent. Clinical information, including epilepsy type and etiology, seizure frequency, disease duration, medication history, comorbidities, and treatment modifications during monitoring, was extracted from medical records.

The inclusion criteria for all participants were: (1) age ≥18 years, and (2) no history of cardiovascular disease or use of cardiovascular medications. The study was approved by the Institutional Ethics Committee at the University of Erlangen-Nürnberg.

### 2.2. Laboratory Protocol (Controls)

Healthy participants wore the Empatica E4 wristband on their non-dominant wrist while simultaneous laboratory recordings were conducted after providing written informed consent. They performed a 7-min resting baseline and a 5-min metronomic breathing condition (six breaths per minute), with event markers set to synchronize the devices.

Laboratory measures included three-lead electrocardiography (ECG; 200 Hz) to derive R-R intervals (RRIs) and continuous systolic and diastolic blood pressure (SBP and DBP) using noninvasive finger pulse photoplethysmography (Portapres^®^, TPD Biomedical Instrumentation, Amsterdam, Netherlands). Data analysis was conducted using a custom-designed system (SUEmpathy™, SUESS Medizin-Technik GmbH, Aue, Germany).

### 2.3. Clinical Protocol (Patients)

PWE wore the E4 continuously on their non-dominant wrist for up to five days during their hospital-stay at the Epilepsy Center of the University Hospital Erlangen (Friedrich-Alexander-Universität Erlangen-Nürnberg). Participants could temporarily remove the device if necessary and were instructed to mark these intervals using event markers. For recordings exceeding 24 h, the device was exchanged every 48 h to maintain battery life. To increase the comparability of the data and minimize motion artifacts, the data was analyzed during segments in which an awake EEG was conducted. A daily wake EEG is usually performed during video-EEG monitoring. For this examination, participants rested in a supine position for 30 min and were asked to follow standardized instructions at defined time points, such as opening or closing their eyes. Periods during which participants fell asleep were excluded from the analysis for the evaluation of wake states. The recordings were segmented into five-minute intervals, and the mean value of all Empatica E4 signals was calculated for each interval. If a participant did not have a wake EEG, five-minute EEG segments of relaxed wakefulness were identified instead. These intervals were averaged and included in the statistical analysis. For sleep analysis, an epileptologist identified 15 min each of N2, N3, and REM sleep per night for each participant and classified them according to American Association of Sleep Medicine (AASM) guidelines. Each 15-min epoch was divided into three five-minute segments. Mean values for Empatica E4 signals were calculated for each segment, consistent with the procedure applied during the wake EEG analysis. Data were downloaded after removal.

### 2.4. Empatica E4

The Empatica E4 wristband includes four sensors: Photoplethysmography (PPG) to derive blood volume pulse (BVP), interbeat interval (IBI), heart rate (HR), and heart rate variability (HRV). Electrodermal activity (EDA) is measured by two electrodes that detect changes in skin conductance. A 3-axis accelerometer captures movement. An infrared thermopile measures skin temperature [15].

### 2.5. Data Preprocessing and Analysis

According to the Task Force of the European Society of Cardiology and the North American Society of Pacing and Electrophysiology (1996) [16], five-minute recordings are sufficient to reliably assess HRV. Furthermore, recent findings indicate that the E4 can accurately estimate pulse rate in recording intervals as short as 10 s, underscoring its potential for flexible clinical applications [11]. For baseline recordings, we used 5 min intervals of PWE and the baseline laboratory measurement for healthy controls. Since metronomic breathing was measured for only 2 min, we used a shorter interval during this parasympathetic challenge. The raw IBI data were imported into Kubios Premium (Kubios Oy, Finland) for HRV analysis using the built-in automatic correction method.

To analyse HRV, we collected

- mean HR in bpm: average heart rate, calculated from the detected interbeat intervals (IBI)
- SDNN: Standard deviation of normal-to-normal (NN) intervals, excluding ectopic or abnormal beats
- RMSSD in ms: the root mean squared differences of successive difference of IBI, also based on normal sinus beats. RMSSD is the main estimation for PNS mediated changes in HRV [17]
- Coefficient of variation (CV in %): a normalized measure of variability relative to the mean
- Evaluation of autonomic cardiovascular modulation comprises not only time- but also frequency domain evaluation, as HR and BP values show slow underlying fluctuations that are largely mediated by undulating activity of the autonomic nervous system. As the current study was designed to determine validity of the Empatica E4 wristband but not to test for autonomic dysfunction in our patient cohort, we did not perform spectral analysis.

Electrodermal activity (EDA, µS) and temperature (°C) signals were recorded at 4 Hz and extracted for analysis using a matlab protocol with minimal preprocessing.

### 2.6. Statistical Analysis

Descriptive statistics, including mean and standard deviation (SD), were calculated for all variables. Intraclass correlation (ICC) was used to assess the agreement between two methods. Cross-correlations (CC) were calculated to determine the relationships between variables, with a threshold of >0.80 considered as valid. Normality was assessed using Kolmogorov–Smirnov tests. Mann–Whitney tests were performed to detect differences between laboratory and E4 recordings. Bland–Altman plots were constructed to evaluate the agreement between the two methods, and 95% limits of agreement (LoA) were calculated to estimate the interval where the true value is expected to fall for 95% of the differences. As this was an exploratory study, significance level was set at *p* < 0.05, without correction for multiple testing.

## 3. Results

### 3.1. Comparison of Empatica E4 and Laboratory HRV Measurements in Healthy Controls During Rest and a Parasympathetic Activation Maneuver (Metronomic Breathing)

Between baseline (BL) and metronomic breathing (MB), both the laboratory device and the Empatica E4 demonstrated significant increases in RMSSD (ms), CV (%), and SDNN (ms), reflecting the expected enhancement of parasympathetic activity during controlled breathing. Although the E4 recorded systematically higher absolute RMSSD values than the laboratory device (*p* < 0.001), both systems captured comparable trends across conditions (Table 1).

We compared the baseline measurements in healthy participants derived from the laboratory analyses and the Empatica E4. Agreement analyses demonstrated excellent concordance between the laboratory device and the Empatica E4 for heart rate (HR, bpm) (ICC = 0.93), coefficient of variation (ICC = 0.84), and the standard deviation of RR intervals (ICC = 0.83), indicating that the wearable device provides highly comparable measurements for these parameters. In contrast, RMSSD showed only moderate agreement (ICC = 0.65), with systematically higher values recorded by the E4 compared to the laboratory system. Bland–Altman analyses further highlighted this discrepancy, revealing wider limits of agreement for RMSSD than for the other HRV metrics (Table 2).

### 3.2. Comparison of Wake and Sleep Stages in PWE

Analysis of ANS activity in PWE across sleep stages, as measured by the Empatica E4, revealed significant changes compared to wakefulness. As expected, the mean heart rate was significantly lower during N2, N3, and REM sleep than during wakefulness (all *p* < 0.001). Although differences did not reach statistical significance, parasympathetic activity, as reflected by RMSSD, showed a trend toward higher values during sleep. The coefficient of variation (CV) increased modestly in N2 and REM sleep compared to wakefulness. Mean peripheral skin temperature (in °C) was significantly higher during all sleep stages compared to wakefulness (all *p* < 0.001). Electrodermal activity (EDA µS) showed no significant differences across sleep stages (Table 3).

### 3.3. Comparison of PWE with and Without Intake of Sodium Channel Blockers

We compared PWE who were treated with sodium channel blockers, such as lamotrigine, oxcarbazepine, or lacosamide, to those who were not taking these medications. Intake of sodium channel blockers was associated with significant alterations in autonomic regulation. Compared to patients not taking these medications, sodium channel blocker induced a higher mean heart rate (*p* = 0.005), reduced RMSSD (*p* = 0.019), lower CV (*p* = 0.021), and reduced SD RR (*p* = 0.027). These results suggest an overall reduction in vagal activity. However, no significant group differences were found for skin temperature or electrodermal activity (Table 4).

## 4. Discussion

Our results suggest that the Empatica E4 reliably estimates HRV compared to laboratory standards, particularly under resting conditions, although caution is warranted when interpreting absolute RMSSD values. In PWE, we captured distinct changes in autonomic regulation across sleep stages, including reduced heart rate and increased temperature during non-rapid eye movement (NREM) sleep. These changes are consistent with enhanced parasympathetic activity. Furthermore, the reduction in HRV observed under sodium channel blockers suggests a measurable drug effect on autonomic balance, which could be relevant for individual risk stratification in epilepsy. These results underscore the Empatica E4′s potential as a practical tool for autonomic function monitoring in controlled and real-life clinical settings. Our study adds to earlier research that it evaluates the device’s performance in a clinical population (PWE) in a hospital setting and explores its ability to detect ANS modulation induced by sleep and sodium-channel-blocking ASM.

In line with our findings, previous studies have demonstrated strong correlations between HRV measurements obtained with the Empatica E4 and those obtained with validated laboratory devices [8,18,19,20,21,22]. Generally, agreement of HRV parameters is higher during rest than during activity [18,19,20]. In our study, the E4 delivered higher RMSSD values, but this fact merely compromises validity of the findings because RMSSD was analysed comparatively rather than against normative cut-offs. RMSSD is considered a robust, relatively artefact-resistant parameter, and it is recommended for HRV assessment [7]. However, PPG-based methods, like those derived from the Empatica E4, tend to produce higher RMSSD values than ECG-based systems. This is likely because PPG measures both cardiac rhythm and vascular modulation, whereas ECG measures only cardiac electrical activity. The inclusion of vascular variability adds to indices like RMSSD, which explains the consistently higher values from PPG-based devices like the Empatica E4. Studies by Diehl et al. and Schuurmans confirm this finding, reporting strong correlations alongside consistently higher absolute RMSSD values [8,23].

In our study, sodium channel blockers included oxcarbazepine, eslicarbazepine, lamotrigine, and lacosamide. However, most people with epilepsy (PWE) took combinations of various antiseizure medications (ASM) because we recruited patients at a tertiary epilepsy center with a high percentage of medically refractory epilepsy. Although decreases in RMSSD under sodium channel blockers like carbamazepine have been reported [24], data on this subject is scarce. In order to exclusively assess possible HRV alterations, that can be ascribed to one pharmacological pathway, i.e., sodium channel blockage, we did not include patients with other anti-seizure medication (such as carbamazepine), and rather focused on this widely used class of drugs.

The parasympathetic activation reflected by an increase in HRV during sleep seems expected, and represents physiologically changes [25]. Since ANS function during sleep was not assessed in healthy controls, it is unclear whether the observed parasympathetic activation in PWE is comparable to or diminished relative to that in healthy controls. An aspect, that should be addressed in future studies. The increase in peripheral skin temperature is also expected and in line with a previous study [26].

Our study is further limited by several other aspects. First of all, it was limited by the small sample size. The subgroups of PWE for sodium channel blocker analyses were particularly small, which may limit statistical power and generalizability. Only six PWE were not taking any antiseizure medication (ASM), and most were on polytherapy (≥2 ASMs). We did not examine potential effects of individual ASMs or polytherapy beyond sodium channel blockers. Moreover, factors related to epilepsy severity, such as seizure frequency, occurrence of generalized tonic–clonic seizures, or duration of epilepsy, were not considered. These variables could represent relevant confounders influencing autonomic parameters; however, due to the limited sample size, we restricted our analyses to maintain statistical validity. Further, we did not evaluate clinical endpoints such as SUDEP incidence. Although measuring HRV via a wearable device like Empatica E4 is promising, our study shows limitations in measuring RMSSD values and signals beyond HRV.

Finally, a major limitation of the present study is that both EDA and temperature data were only recorded with the E4 without being compared to established methods. In contrast to HRV, reliability of EDA measurements with the Empatica E4 is limited. Several validation studies have reported reduced EDA accuracy compared with finger-based measurements [18,19,20,21]. Few studies have assessed temperature measurements. For instance, Xu et al. reported that intra- and postoperative temperatures measured with the E4 were, on average, 2.2 °C lower than their internal standard with high variability [27]. Thus, EDA and temperature data should be interpreted with caution, even though the observed temperature increase during metronomic breathing and during sleep aligns with the expected activation of the parasympathetic nervous system.

Our findings highlight the clinical potential of wearable devices, such as the Empatica E4, for detecting autonomic dysfunction in people with epilepsy. Since autonomic dysregulation is a primary mechanism of SUDEP, continuous HRV monitoring with unobtrusive devices could facilitate early risk stratification and personalized prevention strategies [28,29]. Emerging evidence supports the role of wearable seizure detection devices in SUDEP prevention. In the context of detecting tonic–clonic seizures, multi-modal wearables have been shown to facilitate continuous, unobtrusive monitoring that enables caregiver response during seizures—even when the patient is unattended—helping prevent injury and potentially SUDEP [30,31]. Beyond long-term monitoring, wearables may also be valuable in emergency settings where the rapid, noninvasive detection of autonomic imbalance could guide acute management. As wearable technology becomes more integrated into epilepsy care, our results contribute to the growing body of evidence showing that such devices can bridge the gap between research, routine clinical practice, and real-world SUDEP prevention.

## 5. Conclusions

This study demonstrates that the Empatica E4 is a reliable tool for measuring HRV. Yet, further studies are needed to confirm whether wearable devices can possibly detect clinically meaningful changes in ANS function beyond our findings during sleep and under sodium blocker channels. HRV is the most robust and clinically relevant parameter compared to electrodermal activity or temperature. The E4 showed good concordance with the gold standard of laboratory devices and was sensitive to the expected patterns of ANS modulation during physiological states such as sleep and pharmacological influences, e.g., treatment with a sodium channel blocker. Although short-term measures of HRV, such as 5 min, are not comparable assessments from a 24-h recording, our study adds to earlier research that it evaluates the device’s performance in a clinical population in a hospital setting. These results suggest the E4′s potential as a practical tool for assessing autonomic regulation in ambulatory and clinical settings, especially when standardized autonomic testing is unavailable.

Although Empatica discontinued the E4 in favor of the more advanced EmbracePlus device in early 2025, our results emphasize the broader utility of wearable, photoplethysmography (PPG)-based systems for continuously monitoring autonomic function. Such technologies may be especially valuable in the context of epilepsy, where autonomic dysfunction contributes to morbidity and the risk of sudden unexpected death in epilepsy (SUDEP). These devices could complement traditional clinical assessments, inform treatment decisions, and provide additional insights into emergency settings by enabling unobtrusive, real-world monitoring. Future studies with larger, more diverse cohorts that incorporate next-generation wearables and long-term follow-up are essential to establishing their prognostic value and clarifying their role in epilepsy management and SUDEP risk stratification.

## Figures and Tables

**Table 1:** Baseline (BL) vs. Metronomic breathing (MB) in healthy controls: Empatica E4 vs. laboratory (N = 29).

Device	Parameter	*p*-Value	BL Mean (±SD)	MB Mean (±SD)
**Lab**	HR (bpm)	0.500	70.99 ± 8.19	71.40 ± 7.44
	RMSSD (ms)	<0.001	33.09 ± 16.31	46.76 ± 22.51
	CV (%)	<0.001	5.37 ± 2.39	9.44 ± 3.52
	SDNN (ms)	<0.001	45.44 ± 20.97	78.47 ± 29.80
**Empatica E4**	HR (bpm)	0.774	71.35 ± 8.52	70.87 ± 7.24
	RMSSD (ms)	<0.001	43.97 ± 16.81	53.13 ± 20.28
	CV (%)	<0.001	5.60 ± 2.37	8.31 ± 2.92
	SDNN (ms)	<0.001	47.15 ± 20.10	70.84 ± 27.41

BL baseline, bpm beats per minute, CV coefficient of variation, HR heart rate, MB metronomic breathing, RMSSD root mean squared differences of successive difference of intervals, SD standard deviation, SDNN standard deviation of the normal to normal interval.

**Table 2:** Comparison of laboratory vs Empatica E4 in healthy controls (n = 29).

	*p*-Value	Lab	E4	ICC	CC	r	LoA
**HR (bpm)**	0.41	70.99	71.35	0.93	0.93	0.93	−49.06–57.42
**RMSSD (ms)**	<0.001	33.09	43.97	0.65	0.79	0.79	−32.03–10.27
**CV (%)**	0.35	5.37	5.60	0.84	0.84	0.84	−2.84–2.37
**SDNN (ms)**	0.45	45.44	47.15	0.83	0.83	0.83	−25.31–21.90

Bpm beats per minute, CC correlation coefficient, CV coefficient of variation, HR heart rate, ICC intraclass correlation coefficient, LoA limits of agreement, RMSSD root mean squared differences of successive difference of intervals, SDNN standard deviation of the normal to normal interval.

**Table 3:** Comparison of ANS modulation during different sleep stages to wakefulness in PWE as measured by Empatica E4.

Parameter	Awake (Mean ± SD)	N2	N3	REM (Mean ± SD)	*p*-Value	*p*-Value	*p*-Value
		(Mean ± SD)	(Mean ± SD)		(Awake vs N2)	(Awake vs N3)	(Awake vs REM)
**HR (bpm)**	73.46 ± 15.04	64.76 ± 11.17	64.14 ± 10.33	63.88 ± 9.96	<0.001	<0.001	<0.001
**RMSSD (ms)**	46.42 ± 27.80	54.47 ± 33.30	52.91 ± 30.72	59.12 ± 38.42	0.057	0.165	0.051
**CV (%)**	0.06 ± 0.04	0.07 ± 0.05	0.07 ± 0.04	0.08 ± 0.05	0.007	0.098	0.009
**SDNN (ms)**	3.25 ± 1.41	3.32 ± 1.12	2.73 ± 0.98	0.89 ± 0.86	0.836	0.086	0.505
**Temp (°C)**	33.59 ± 1.34	34.85 ± 0.68	35.05 ± 0.78	34.83 ± 0.70	<0.001	<0.001	<0.001
**EDA**	0.95 ± 1.33	0.89 ± 0.83	1.20 ± 1.51	3.03 ± 0.84	0.859	0.533	0.574

CV coefficient of variation, EDA electrodermal activity, HR heart rate, RMSSD root mean squared differences of successive difference of intervals, SD standard deviation, SDNN standard deviation of the normal to normal interval, Temp temperature.

**Table 4:** ANS modulation under sodium channel blockers (N = 27 vs. no sodium channel blockers, N = 15) in PWE.

Parameter	*p*-Value	No Blockers vs. Sodium Channel Blockers (Mean ± SD)
**HR (bpm)**	0.005	65.43 ± 9.11 vs. 77.80 ± 14.94
**RMSSD (ms)**	0.019	63.48 ± 33.47 vs. 38.07 ± 22.31
**CV (%)**	0.021	0.081 ± 0.012 vs. 0.047 ± 0.031
**SDNN (ms)**	0.027	3.52 ± 1.65 vs. 3.01 ± 1.21
**Temp (°C)**	0.336	33.38 ± 0.97 vs. 33.82 ± 1.46
**EDA**	0.771	1.13 ± 1.08 vs. 0.99 ± 1.44

ANS autonomic nervous system, CV coefficient of variation, EDA electrodermal activity, HR heart rate, RMSSD root mean squared differences of successive difference of intervals, SD standard deviation, SDNN standard deviation of the normal to normal interval, Temp temperature.

## Data Availability

The raw data supporting the conclusions of this article will be made available by the authors on request.

## Abbreviations

The following abbreviations are used in this manuscript:

ASM	antiseizure medication
BL	baseline
Bpm	beats per minute
CC	correlation coefficient
CV	coefficient of variation
EDA	electrodermal activity
HR	heart rate
ICC	intraclass correlation coefficient
LoA	limits of agreement
MB	metronomic breathing
PWE	person with epilepsy
RMSSD	root mean squared differences of successive difference of intervals
SD	standard deviation
SDNN	standard deviation of the normal to normal interval
Temp	temperature
